# The Cellular and Molecular Carcinogenic Effects of Radon Exposure: A Review

**DOI:** 10.3390/ijms140714024

**Published:** 2013-07-05

**Authors:** Aaron Robertson, James Allen, Robin Laney, Alison Curnow

**Affiliations:** 1 Clinical Photobiology, European Centre for Environment and Human Health, University of Exeter Medical School, University of Exeter, Knowledge Spa, Royal Cornwall Hospital, Truro, Cornwall TR1 3HD, UK; E-Mails: james1.allen@exeter.ac.uk (J.A.); a.curnow@exeter.ac.uk (A.C.); 2 Clinical Oncology, Sunrise Centre, Royal Cornwall Hospital, Truro, Cornwall TR1 3LJ, UK; E-Mail: robin.laney@rcht.cornwall.nhs.uk

**Keywords:** radon, carcinogenesis, cytogenetics, DNA damage, alpha particles, bystander effect, chromosome aberrations, micronuclei, linear, no-threshold, hormesis

## Abstract

Radon-222 is a naturally occurring radioactive gas that is responsible for approximately half of the human annual background radiation exposure globally. Chronic exposure to radon and its decay products is estimated to be the second leading cause of lung cancer behind smoking, and links to other forms of neoplasms have been postulated. Ionizing radiation emitted during the radioactive decay of radon and its progeny can induce a variety of cytogenetic effects that can be biologically damaging and result in an increased risk of carcinogenesis. Suggested effects produced as a result of alpha particle exposure from radon include mutations, chromosome aberrations, generation of reactive oxygen species, modification of the cell cycle, up or down regulation of cytokines and the increased production of proteins associated with cell-cycle regulation and carcinogenesis. A number of potential biomarkers of exposure, including translocations at codon 249 of *TP53* in addition to *HPRT* mutations, have been suggested although, in conclusion, the evidence for such hotspots is insufficient. There is also substantial evidence of bystander effects, which may provide complications when calculating risk estimates as a result of exposure, particularly at low doses where cellular responses often appear to deviate from the linear, no-threshold hypothesis. At low doses, effects may also be dependent on cellular conditions as opposed to dose. The cellular and molecular carcinogenic effects of radon exposure have been observed to be both numerous and complex and the elevated chronic exposure of man may therefore pose a significant public health risk that may extend beyond the association with lung carcinogenesis.

## 1. Introduction

**Figure 1:**
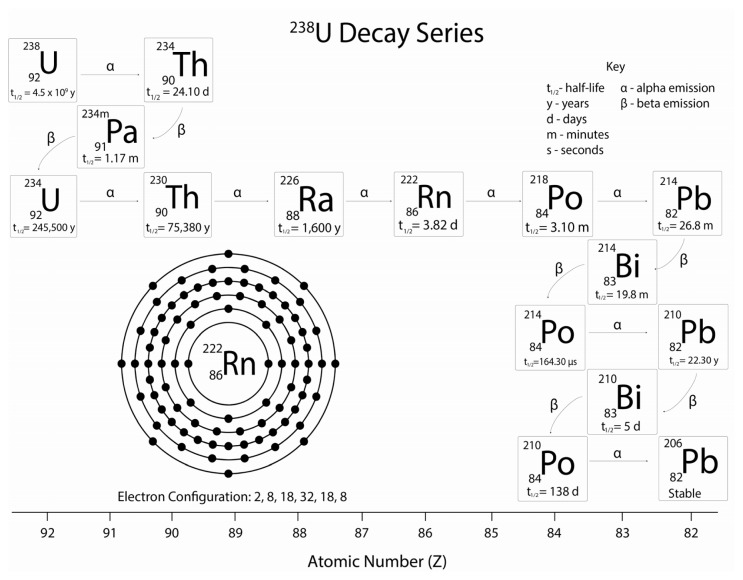
The decay series of uranium-238 indicating its decay products, their half-lives and the electron configuration of radon-222 complete with each respective atomic number.

Radon-222 (further referred to as radon) is a naturally occurring inert gas formed in the decay series of uranium-238 (Figure 1), which can be found in trace amounts in many rocks and soils. As radon decays, it produces radioactive progeny and emits significant levels of alpha radiation, along with lower levels of beta and gamma radiation, of various energies (Table 1), leading to biological damage that can be dangerous to human health [1,2]. Radon levels can vary greatly depending upon a number of factors, including geographical location, temperature and geology [3]. A link between radon and lung carcinogenesis has already been established and radon is thought to be the second leading cause of lung cancer in the UK after smoking [4–10], with evidence of a synergistic effect between radon and tobacco smoke [7]. Greater than 50% of the average annual background radiation dose is due to radon and its decay products [11], which, due to electrostatic forces, can attach to aerosols [12,13] and plateout on the skin [14–16] significantly increasing the potential dose delivered to this organ.

Radioactive decay occurs in unstable atomic nuclei with the emission of ionizing particles resulting in a release of energy. With a half-life of 3.82 days, radon can accumulate in buildings before rapidly decaying into lead-210 with a half-life of 22 years. Other isotopes of radon, such as radon-219 (actinon) and radon-220 (thoron), are also present in the natural environment and a number of studies have suggested that thoron may also be detrimental to human health [17–21]. Nevertheless, they are often left unacknowledged when investigating potential health effects in humans due to their shorter half-lives of 4 and 56 s, suggesting harmful accumulation is unlikely as a result of reduced exposure estimates [18–20]. The lack of acknowledgement of thoron is of interest when considering Thorotrast. Thorotrast is a medically administered radioactive contrast agent that was used throughout the 1930s and 1940s and is strongly associated with liver carcinogenesis as a result of chronic internal radiation exposure [22,23]. Of particular intrigue is that Thorotrast treated patients are known to exhale elevated levels of thoron and there have been some investigations into the potential for patients to develop lung cancer although the studies remain inconclusive [24–28].

Alpha particles represent the predominant form of radiation emitted as a result of the decay of radon. Despite their limited tissue penetration capability, alpha particles can cause significant biological damage in exposed tissue due to their high relative biological effectiveness (RBE) [29]. Beta and gamma-radiation are also emitted from the decay of radon progeny, however the RBE compared to alpha particle ionization is minimal [30] (Table 1). Alpha particles consist of a helium nucleus (two protons and two neutrons) and have the potential to deposit large amounts of energy as they traverse matter. In comparison to beta particles (electrons) and gamma radiation (photons) they are described as having a high linear energy transfer (LET). Mainly as a result of this high-LET classification, alpha particles are more biologically significant than either beta or gamma radiations, reacting much more readily with DNA and generating oxidative stress through radiolysis despite their reduced penetrating capability. Tissue regions and cell types that are within depths traversable by alpha particle exposure can be particularly susceptible to biological damage. The most substantial alpha emitters from radon decay are polonium-218 (6.0 MeV) and polonium-214 (7.69 MeV) and have penetration depths of 47 μm and 70 μm respectively [29], suggesting high levels of irradiation, particularly of the bronchial epithelium and at bifurcation sites, when inhaled into the lungs [31–35].

It has previously been assumed that the depth of skin would be too thick for alpha particles to successfully penetrate and provide sufficient exposure to the sites vulnerable to mutagenesis. However, research now suggests that exposure to areas of the skin that are particularly thin, such as on the face where epidermal thickness has been measured to be 10–40 μm [36] could result in significant exposure of the basal layer to alpha radiation [21,36–38], theoretically increasing the likelihood of the potential for biological damage. The South-West of England has both the highest rates of non-melanoma skin cancer (NMSC) and the highest average radon concentrations in the UK, with epidemiological evidence now suggesting that in Devon and Cornwall increased domestic residential radon exposure may be a risk factor for the development of squamous cell carcinoma of the skin [39,40]. It should be noted that a number of other cancers have also been suggested to have an increased risk with high radon concentrations including leukemia [41,42] and gastrointestinal malignancies [43,44], although any evidence of a cause-effect relationship remains conjectural [10,45,46].

Ionizing radiation in the form of alpha particles can cause DNA damage from chromosomal aberrations [47], double strand DNA breaks and generate reactive oxygen species (ROS) [48], resulting in cell cycle shortening, apoptosis and an increased potential for carcinogenesis.

Radon concentrations are typically tested using alpha track detectors usually placed in multiple sites throughout the home or workplace to obtain an applicable result that takes into account the variability between rooms. As a result of the heterogeneous distribution of uranium in rocks and soils around the world, radon concentrations can vary significantly between different locations. Not only can there be variation in concentrations but the levels that are deemed acceptable also vary between different countries. In the UK, the radon action level (the threshold whereby action should be taken to reduce radon concentrations) is deemed to be 200 Bq·m^−3^ (10 times the national average of 20 Bq·m^−3^) and in the US this figure is lower at 148 Bq·m^−3^ (4 pCi/L). If the concentrations of these figures are observed in domestic or work environments it is recommend that remedial action should be taken to reduce the risk (risk estimates for lung cancer are displayed in Table 2). However, there is an inherent difficult in accurately predicting or determining personal exposure dosimetry based upon values derived using alpha track detectors as often individuals can spend varying amounts of time in areas away from those that have been measured. In response, a number of studies have tried to derive a more accurate quantification of exposure through the use of plastics and other materials that a sensitive to tracks produced by alpha particles with examples including eye-glass lenses [49], passive personal dosimeters [50] and wrist watch detectors [51]. Nevertheless, accurate exposure estimates to the general population can often remain difficult to accurately obtain and the requirement for cheap, portable and reliable personal dosimeters has been acknowledged [51] and until such devices are routinely employed across multiple *in vivo* studies the effective comparison of exposures between laboratory based biological studies and those at physiological exposures will remain difficult to compare effectively.

The employment of radon spas, whereby radon exposure is used therapeutically for pain relief, has not only been conducted historically but has also persisted despite both the recognition of radon as a carcinogen and the continuing debate regarding safe levels of exposure [52]. Typical sources used by radon spas include natural springs, thermal pools or enclosed environments in the presence of radium-rich rocks for which all are located in areas with very high radon concentrations. Measured levels can reach thousands of Bq·m^−3^. These high exposures have raised a number of health issues not just for the individuals that are treated but also for workers at the spas [53]. Regardless of the potential carcinogenic risk of radon spa use, decreases in perceived pain relief and increases in joint mobility have been reported in rheumatoid arthritis sufferers following treatment [54].

Much of the evidence obtained relating to radon’s carcinogenicity has been from studies performed on cohorts of uranium miners that have historically received high levels of radon exposure. Concentrations of radiation in mines are often reported in terms of working levels whereby one working level (WL) is defined as the alpha particle emission of 1.3 × 10^5^ MeV from radon and its daughters. One working level month (WLM) represents this value over the length of working time in a single month, averaged to 170 h, *i.e.*, 1WL × 170 h. The miner cohorts have often contained incomplete data with regards to the lifestyle conditions of the miners, which has resulted in a difficulty in identifying implications at lower, more archetypal, exposures. However, a number of cytogenetic studies have revealed cellular and molecular mechanisms that play a crucial role in the elucidation of radon’s neoplastic potential and they can provide an approach to investigate any harmful effects that may affect mutation or cancer incidence as a result of environmental radon exposure. The aim of this review is to provide a summary of these developments and to investigate the evidence of a mutagenic effect of radon at low exposure levels. A significant proportion of the current literature employs alpha particle emitters as surrogates in place of radon and its daughter products and so the cellular and molecular effects of alpha emitters such as polonium-238 or americium-241 (that are often employed to imitate the effects of radon *in vitro*) are included alongside the effects produced by radon itself.

## 2. Genetic Effects

### 2.1. Environmental Investigations at the *TP53* Locus

#### 2.1.1. Hotspot Mutations of the *TP53* Gene

The tumor suppressor gene *TP53* (previously named *p53*), located in humans on chromosome 17p13.1, codes for the p53 protein and is part of a network that is integral to the effective maintenance of the cell cycle. *TP53* mutations and deletions [56] have been frequently observed in various cancers, including those of the lung, and investigations have previously located unique mutations in regions referred to as hotspots that could result from radon exposure, although evidence remains conjectural. A number of *TP53* mutations are associated with tobacco smoking-induced lung cancers and similar mutation hotspots have been identified that are not associated with other types of cancer, e.g., codon 157 [57]. These mutation spectra are also different between smokers and non-smokers [58,59]. If it was possible to identify hotspot regions for radon-induced lung cancers in a similar manner, this could help to provide a unique biomarker that contributes to the understanding of the aetiology of the disease and contribute to the elucidation of risk at typical exposure levels. A number of studies have explored this prospect (Table 3).

#### 2.1.2. Investigations of *TP53* Mutations in Uranium Miners

Vähäkangas *et al.* [60] studied 19 lung cancers from New Mexican uranium miners and identified 9 various *TP53* mutations, of which none were the G:C to T:A transversions known to be associated with tobacco smoke exposure [61]. Furthermore, a study of 52 large and squamous cell carcinomas of the lung diagnosed in Colorado uranium miners, found that 31% possessed the matching AGG to ATG (Arg→Met) transversions, but at the second base pair region of codon 249 in exon 7. This was also observed in 3 of 5 cancers from non-smoking miners [62], leading the authors to highlight this region as a potential biomarker for radon associated lung cancer which, they suggest, is due to the rarity (0.4%) of this specific mutation occurring insmoking-associated lung cancers, although further studies have not been able to show a similar association. A follow up study on the same cohort of miners investigated adenocarcinomas as opposed to the original squamous cell carcinomas and failed to demonstrate the codon 249 transversion previously observed [63].

The analysis of archived tumors from 50 uranium miners from the German Wismut company archive cohort [64] found no evidence for the codon 249 hotspot in adenocarcinomas, squamous, large cell, small cell or mixed lung carcinomas [65]. A follow-up study providing an additional genetic analysis of the cohort of exposed uranium miners identified only 2% (1 of 50) of the studied tumors displayed the G to T mutation in codon 249 [66]. An additional study analysed a greater proportion of the squamous cell carcinomas from the same Wismut company cohort, exploring the speculation that the radon induced hotspot may only be an indicator of radon associated exposure in squamous cell carcinomas. Of the 29 squamous cell carcinomas studied, two demonstrated the G to T mutation leading the authors to conclude that the hotspot is not a likely marker for radon exposure in squamous cell carcinomas [67].

Popp *et al.* [68] studied the lung cancers of 16 uranium miners (12 squamous cell carcinomas, 3 adenocarcinomas and 1 small-cell carcinoma) in Germany and 13 non-mining patients who had lung cancer (4 squamous cell carcinomas, 8 adenocarcinomas and 1 large cell carcinoma) and were all from the same geographical region. In line with many of the previous studies, they found no codon 249 G to T transversion in any of investigated patients, with the exception of the only non-smoking uranium miner present in the study. However, they did identify a codon 213/3 polymorphism in 19% of the miners and 8% of the non-miners.

It could therefore be concluded that, to date, no clear radon-induced *TP53* hotspot mutation has been identified (other than in the Colorado study [62]) in uranium miner cohorts.

#### 2.1.3. Investigations of *TP53* Mutations in Non-Mining Individuals

Lo *et al.* [72] analysed a total of 17 lung cancers of individuals from Devon and Cornwall, UK, with homes of known radon concentrations grouped into high radon exposure (greater than 200 Bq·m^−3^), low exposure (less than 20 Bq·m^−3^) or intermediate exposure. Three lifelong non-smokers were included, of which two were in the intermediate group and one was in the high exposure group. No evidence was found in any of the studied cases for the *TP53* mutation hotspot at codon 249 from either the 14 smokers’ or 3 non-smokers’ lung cancers. The authors suggest that this may be due to a much lower radiation level compared to the Taylor *et al.*, [62] study and that this may either represent a dose-response relationship or, along with other authors [73], may be the result of mycotoxins from the Colorado mines. A similar study of individuals from homes of known radon concentration, where at least 20% were greater than 148 Bq·m^−3^, in Galicia, Spain, analysed 72 lung cancers and found no correlation between variation in radon exposure and either *TP53* mutations or p53 expression, although the number of sequenced samples for mutation analysis was small (*n* = 4) with a relatively low radon concentration of between 11.1 and 51.8 Bq·m^−3^[71].

A nationwide population-based study in Sweden including 83 non-smoking and 250 smoking-induced lung cancers investigated the association between *TP53* mutations and time-weighted average radon concentrations of less than 50 Bq m^−3^ or greater than 140 Bq·m^−3^ adjusting for smoking status, age and gender [70]. A final analysis of 243 tumors determined a *TP53* mutation rate of 23.9%, whereby a higher percentage of individuals with *TP53* mutations were observed in the high residential radon exposure group (42.9%) compared to the lower exposure group (20.9%), particularly among non-smokers. It should be noted, however, that no specific difference in mutation pattern was observed between groups and while information on smoking habits was obtained from questionnaires, the authors drew attention to the uncertainties relating to the measurements and previous estimates of the individuals’ radon exposure.

In further work by Vähäkangas *et al.* [57], analysis of 126 lung cancers (including adeno, broncho-aleveolar, squamous, small cell and mixed carcinomas) from ex-smoking (*n* = 9) and non-smoking (*n* = 117) women, aged between 41 and 84 at the time of diagnosis, demonstrated a higher *TP53* mutation frequency in ex-smokers (67%) than in non-smokers (19%), which, as the authors suggest, is due to the persistence of molecular damage even 15 years after smoking cessation. Non-smokers were defined as having neither smoked more than 100 cigarettes nor having used any tobacco products for longer than 6 months during their lifetime. Ex-smokers were defined as having stopped smoking at least 15 years before the study. Radon measurements were performed on the homes that each individual had occupied for at least 1 year during the preceding 30 years [74] and although the authors identified a *TP53* mutation difference between lung cancers from smokers and non- or ex-smokers (most visibly a lower mutation frequency in non-smokers compared to smokers), they found no statistically significant relationship with *TP53* mutations and radon exposure.

Again, it appears that, similarly to the mining cohorts, no consistent *TP53* mutation pattern associated with radon exposure has been identified in non-mining cohorts.

### 2.2. *TP53* Laboratory Investigations

*In vitro* exposure of cultured A549 cells to alpha particles produced by Pu-238 (with an activity of 2.7 Mbq) ensuring a dose of 0.6 Gy (with a rate of 1 Gy per minute) using a specially constructed 45 mm diameter culture dish with 1.5 μm mylar film bases resulted in a significant increase in the production of p53, and rats that have been exposed *in vivo* to radon exhibit an increase in the number of G_1_ phase cells with an accompanying decrease of those in S phase [75], suggesting an augmentation of cell cycle arrest. Normal human bronchial epithelial cells (NHBE), cultured from a single donor, exposed to 4 Gy (approximately equivalent to 1460 WLM in a uranium mine) of high-LET alpha radiation (using a Pu-238 source) demonstrated no G to T transversions at the *TP53* hotspot, although there was evidence for G to A transitions at codon 249 and a C to A transversion at codon 250 [69]. The authors note that this is in agreement with the study by Vähäkangas *et al.* [60] and suggest the high frequency of the G to A transition is indicative of alpha particle exposure, although they underline the limitations of the single donor origin and propose that observations may exhibit different results from other donors.

Cellular exposure has also demonstrated a malignant transformation potential following a single exposure (0.6 Gy) to alpha particles [76]. Telomerase-immortalised human prostate epithelial (HBPE) cells retaining morphology, contact growth inhibition and anchorage dependency [77] were malignantly transformed following helium-4 exposure and insertion into SCID (severe combined immunodeficiency) mice. The resulting tumors were characterised as adenocarcinomas, with mutations and deletions of the *TP53* gene including: G to T transversions at codon 314 (Pro→Leu) and 383 (Ala→Ser) and a C to T transition at codon 1090 (Pro→Leu) of exons 4–10 [76]. However, the study did not detect a codon 249 hotspot mutation.

Belinsky *et al.* [78] identified that exposure of F344 rats to the alpha emitter Pu-239 did not sufficiently suggest that the *TP53* mutation underlies the aetiology of the genesis of rat lung tumors with just 7% (2/29) of rat squamous cell carcinomas representing alterations of the *TP53* gene, both of which were G to A transitions at codons 280 and 283. In a similar study of tumors from radon progeny exposed F344 rats, direct sequencing revealed 14% (4/29) of the tumors had *TP53* mutations or deletions, including two A to G transitions at codons 124 and 134, homologous to human codons 126 and 136, respectively [79]. No codon 249 hotspot was observed. The authors suggest that inactivation of the *TP53* gene is rarely involved in radon-induced rat lung carcinogenesis due to the infrequent observation of mutations from this study.

It therefore seems evident that although some radon induced *TP53* mutations have been observed in the environmental studies, they have been found on the whole to be infrequent and not associated with a potential codon 249 hotspot mutation.

### 2.3. Environmental Investigations at the *HPRT* locus

The X-linked hypoxanthine phosphoribosyl transferase (*HPRT)* gene encodes a transferase enzyme, found in all cells, which catalyses the conversions of both hypoxanthine to inosine monophosphate and guanine to guanosine monophosphate, thus, affording a key role in the purine salvage pathway [80]. A deficiency of the enzyme can result in Lesch-Nyhan syndrome characterised by the overproduction of uric acid [81]. The *HPRT* gene has been widely used as an indicator of mutagenesis both *in vivo* and *in vitro* for a number of reasons. Firstly, as *HPRT* is X-linked, its hemizygosity implies that mutation of both alleles is not necessary to convert a wild type to a mutant and secondly, *HPRT*^+/−^cells are easily selectable in culture. In addition, the entire gene has been sequenced [82] and both a database and software have been produced for mutational analysis [83–85].

Alpha particle-induced *HPRT* mutations from a number of sources, including radon exposure, have been demonstrated. Furthermore, there is evidence from atomic bomb survivors of a dose dependent increase in *HPRT* mutations [86] up to more than 40 years after exposure [87]. An eight-fold increase in *HPRT* mutant frequency has also been observed in cancer patients one week after radiotherapy [88]. It has therefore been proposed that analysis of mutations at the *HPRT* locus could provide a method to differentiate between genetic damage caused by low or high-LET radiation through the identification of a unique “fingerprint” of damage, or biomarker. As a result, the *HPRT* gene has been of substantial interest in radiation research. Although the combined analysis of a number of separate *HPRT* studies reveal mutations that are spread widely across the gene, there is still some evidence that some regions do appear as potential hotspot sites as found by a study investigating 271 different *HPRT* mutations [89].

#### 2.3.1. Investigations of *HPRT* Mutations in Uranium Miners

A study of a mining cohort from the Radium Hill uranium mine in South Australia [90] found no evidence to suggest radon concentrations affect *HPRT* mutation frequencies compared to a control group of workers located above ground, although they did observe significance for glycophorin A mutation rates. This is of particular interest as a separate study of workers from the same mine concluded that lung cancer mortality of the underground workers was markedly increased compared to the control group [91] and that the difference was unlikely to be due to confounding factors such as smoking.

#### 2.3.2. Investigation of *HPRT* and *BCL-2* Mutations in Non Mining Individuals

The possibility of *HPRT* mutation as a result of domestic radon exposure has been investigated in peripheral T lymphocytes following prior suggestions of an increased risk of acute myeloid leukemia in regions with high radon concentrations [92]. Twenty non-smokers were selected from homes of known radon concentrations in the town of Street, Somerset, UK, where radon concentrations in the selected homes varied by a factor of more than 10. Radon measurements were initially performed for one month before a more detailed follow-up over three months was completed accounting for differences between living room and bedroom concentrations. Regular smokers were excluded from the study on the assumption that they would harbour increased mutation frequencies as a result of tobacco exposure, although three of the included participants did admit to occasional smoking during the previous year. The authors identified a significant association between radon concentrations and *HPRT* mutant frequency using both the first (*p* < 0.01) and second (*p* < 0.05) radon concentration analyses. However, in a follow up study that increased the sample size, blood samples were obtained from 66 of the occupants and analysed for both mutations at the *HPRT* locus and the leukemia and non-Hodgkin lymphoma associated(apoptotic regulator) B-cell lymphoma 2 (*BCL-2*) translocation t(14;18) [93–95]. On this occasion, they observed no significant correlation between radon concentrations and either *HPRT* mutation frequencies or *BCL-2* chromosome translocations [96]. Another investigation of T lymphocytes [97] collected from 120 individuals with varying exposures to radon and tobacco smoke identified a significant translocation frequency at the *BCL-2* locus with a 4.9-fold larger variation in translocation frequency than *HPRT* mutation frequency leading the authors to suggest that *BCL-2* translocations may be a more appropriate indication of cancer risk than *HPRT* mutations due to the dose-dependent increase of *BCL-2* translocations with age [98,99], smoking [100] and because *BCL-2* translocations are already directly associated with a number of cancers (e.g., non-Hodgkin lymphoma).

Despite these studies providing evidence both for and against increased *HPRT* mutant induction after exposure, another study [101] on a separate cohort of 11 individuals with dwellings of known radon concentrations found an inverse relationship between *HPRT* mutations and exposure level, providing a result in deviation from both the Bridges *et al.* [92] and the Liu *et al.* [97] study. In recognition of the small sample size, a follow-up study [102] of 116 different dwellings with concentrations ranging from 50 to 3300 Bq·m^−3^ was performed (all investigated individuals were from houses with a minimum concentration of 100 Bq·m^−3^). The same result was obtained, *i.e.*, an inverse relationship between domestic residential radon exposure and *HPRT* mutation frequency. No association for sister chromatid exchanges (SCEs), chromosome aberrations (CAs) or micronuclei (MN) formation was observed. The authors suggested that exposures to low doses of radon may induce *HPRT* associated repair mechanisms.

A similar lack of a definitive *in vivo* observation of *HPRT* mutation frequencies has also been identified in a study of high school students from Colorado living in homes of known radon concentrations where no correlation between radon exposure and *HPRT* mutant frequency was observed [103]. A study of flight engineers (*n* = 28) exposed to cosmic radiation observed a non-statistically significant increase in *HPRT* mutations, MN formation and CAs [104], with the authors suggesting that the lack of statistical significance of the data may have been due to large individual variations. Although no significant increase was observed, it is interesting to note that the *HPRT* mutations were higher in those subjects with a shorter flight history, which may support the inverse relationship detected by Albering *et al.* [101]. An assessment of factory workers’ exposure to ionizing radiation revealed a correlation between exposure and increased *HPRT* mutant frequency in the lymphocytes of workers who wore dosimeters and were analysed over a 72-week period [105], although no correlation was observed across a shorter time of 36 weeks.

An analysis of Gulf War I veterans exposed to alpha particle emitting depleted uranium has also identified a significant 50% increase in *HPRT* mutant frequency compared to a low exposure group [106] and a number of other studies have also observed the mutagenic and clastogenic potential of depleted uranium exposure [107,108].

The only rat study that has investigated inhaled radon and *Hprt* mutations [109] identified significant increases in both MN formation and *Hprt* mutation frequency in a dose-dependent manner with exposures of 60–120 WLMs across eight hours a day, six days a week. The authors recommended investigating MN and *Hprt* investigations in future studies of the genetic effects of radon exposure.

In line with the previous inconsistencies of the *TP53* environmental and laboratory investigations, it could also be concluded that, to date, no clear radon induced *HPRT* mutation hotspot has been identified and in some cases an inverse relationship between radon and *HPRT* mutation frequency has been observed.

### 2.4. *HPRT* Laboratory Investigations

*In vitro* experiments on T lymphoblasts exposed to alpha particles resulted in an increased *HPRT* mutation frequency along with increased cell death [47]. Alpha particle-induced mutation frequencies were higher when compared to those from X-radiation. Single-hit kinetics best explained the linear nature of the results and using dose, mutation frequency and an approximation of 50,000 genes per cell, they calculated that each alpha particle had the potential to induce approximately 16 mutations per DNA genome.

The exposure of Chinese hamster ovary (CHO) cells to radon and its daughters [110] using a gaseous exposure apparatus [111] of doses between 0.25 and 0.77 Gy identified a linear dose response relationship between dose and induced mutation frequency at the *Hprt* locus, with an induction frequency of 1 × 10^−4^ per viable cell per Gy. When the spectra of mutation types were investigated, there was no significant difference between radon-induced and X-radiation-induced mutants, although a difference was observed between radiation-induced and spontaneous mutations that were not exposed to radiation, with the principal spontaneous-induced mutation resembling a small event not detectable by Southern blot or PCR exon analysis. The principal radiation-induced mutations, which occurred in 48% of mutants, were gene deletions.

While some of these studies demonstrate a certain degree of similarity between low-LET (e.g., X- or gamma-radiation) and high-LET (e.g., alpha-radiation) *HPRT* mutation spectra, suggesting no difference between the two types of exposure, there are further studies that suggest otherwise. Americium-241 exposure of Chinese hamster lung fibroblasts produced a higher fraction of *Hprt* deletions from alpha particle exposure (80%) than X-radiation exposure (60%) [112]. A similar study on human B lymphoblastoid (TK6) cells [113], which employed a different radon dosing apparatus [114], also identified that the predominant *HPRT* mutation resembled a gene deletion with irradiation of 0.15–1.07 Gy producing deletions in 86% of mutants compared to 81% of X-radiation-induced (1.5 Gy) mutations.

Although not directly *HPRT* related, in a follow-up to the Bao *et al.* [113] study, assessment of thymidine kinase (*TK*) gene mutations in TK6 cells revealed 82% of radon exposed mutants possessed a complete gene deletion compared to 74% of those exposed to X-radiation [115], providing evidence in general agreement with the previous study. However, they also observed reduced growth rates in radon exposed mutants compared to X-radiation exposed mutants (an observation also identified in another study [116]) suggesting that radon-induced lesions may be more complex or harder to repair.

Despite the previous studies appearing to be in some agreement with regards to the types of induced damage between high and low-LET, it is evident that such a conclusion may be premature. Further analysis of CHO-K1 cells, following up from the study conducted by Hsie *et al.* [117], failed to detect a difference between *Hprt* mutation spectra [118] and again in a similar contrast to some previous studies, work in human T lymphocytes [119] demonstrated less than 2% total gene deletions at the *HPRT* locus after exposure to radon and its daughters, although partial deletions were still present. Furthermore, human T lymphocytes exposed to high-LET radon (using the same methodology as described by Jostes *et al.* [111]) and low-LET Caesium-137 found that the main marker of low-LET exposed lymphocytes were large deletions of the entire *HPRT* gene and high-LET exposure predominantly resulted in partial deletions and multiple single base changes [120]. Although not comparing high and low-LET exposures, other work in CHO cells using varying doses of Caesium-137 gamma-radiation has observed dose-dependent changes in mutation spectra [121] and a difference in mutation type between exposures of low (resulting mainly in point mutations) and high-dose (resulting in multibase deletion mutations). This suggests that the risks of exposure to low-dose radiation may not be well represented from extrapolations and interpretations of high-dose exposures.

Analyses of the mutation spectra of cells that are not directly irradiated with alpha particles but still receive damage signals from irradiated cells (*i.e.*, “bystander” cells) have shown *HPRT* mutant induction [122], which mainly consist of point mutations compared to a high frequency of deletions in directly irradiated cells [123]. A follow-up study in DNA double-strand repair deficient cells [124] identified a larger bystander effect compared to the control cells, suggesting that an inability for effective DNA repair resulted in increased *HPRT* mutation induction in bystander cells.

Both *in vitro* and rodent studies have demonstrated both similarity and differences between low and high-LET exposures. However, radon is associated with the latter and in this case some deletions and bystander effects have been observed. The results from studies of *HPRT* have often been contradictory and data have been highly variable resulting in a long-running difficulty in elucidating effects of radiation at the *HPRT* locus and in identifying such hotspot regions. Although the *in vivo* studies of individuals living in homes with known radon concentrations coupled with *in vitro* investigations do seem to suggest an association between radon exposure and *HPRT* mutation frequency, it appears logical to conclude that they do not yet seem to have clearly identified a unique region, or hotspot, as a marker of high-LET compared to low-LET exposure. The large variability in reported mutant frequencies and general inconsistencies in reported findings have led some to suggest caution when interpreting the results [103,125] and that *HPRT* mutations may not represent an effective methodology when evaluating cancer risk.

## 3. Chromosome Aberrations

**Figure 2:**
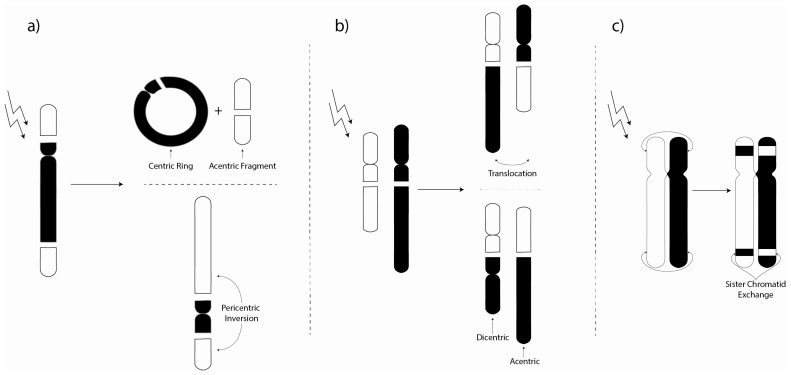
Examples of (**a**) intra-chromosomal aberrations (including pericentric inversions and the formation of centric rings and acentric fragments); (**b**) Inter-chromosomal aberrations (including translocations and the formation of dicentric and acentric fragments); and (**c**) Sister chromatid exchange (whereby genetic material is exchanged between two sister chromatids).

Disruptions to normal chromosomal arrangement represent a major contribution to cellular mutagenicity [126–131]. Cytogenetic analysis has been used for many decades as a tool to determine mutagenic and carcinogenic risks in both domestic and occupational settings and the detection of chromosomal aberrations (CAs) has been associated with numerous chemicals [132–134] and various types of radiation, including both low and high-LET exposures [135–138], in addition to data from atomic bomb survivors [139,140], radiotherapy patients [141–143] and even underground water-well workers [144]. Attempts have also been made to estimate prior exposure through CA analysis [145,146]. The focus here will be placed on reviewing the effects of radon exposure, along with its surrogates, on CAs (Table 4). The cardinal types of structural aberrations include insertions, deletions, translocations, SCEs, ring formation, duplications and inversions (Figure 2). Such structural abnormalities can contribute a significant mutagenic load resulting in a number of identifiable cellular effects (e.g., mitotic delay [147]). For a more complete classification on aberration structures and formations see Savage *et al.* [148] and Bender *et al.* [129].

### 3.1. Environmental Investigations of Chromosome Aberrations

#### 3.1.1. Investigations of Chromosome Aberrations in Uranium Miners

With the progression of cytogenetic techniques, the feasibility of using CAs as a marker of exposure to environmental stressors and of increased cancer risk has been well established. One particular difficulty when using miner cohorts can be ascribing any observed biological effects to a particular exposure, as mining environments are known to contain a number of toxic agents (e.g., arsenic, lead and silica). Despite this limitation, a number of reports have identified an increase in CA frequencies among miners exposed to high radon concentrations, in addition to studies investigating uranium [159] or plutonium [160] compounds.

The analysis of almost 9000 cytogenetic tests from nearly 4000 subjects appeared to determine a significant association between human peripheral blood lymphocyte CA frequency and cancer incidence in radon exposed miners, with just a 1% increase in CAs resulting in a 64% increase in cancer incidence [161]. A follow-up study [162] of a further 1323 tests and 225 individuals provided additional data and found similar results confirming an association between aberration frequency and cancer incidence with a 1% increase in chromosomal aberrations resulting in an increase in cancer incidence of 62%. Of particular note was that an increased frequency of chromatid breaks in 1% of cells resulted in a 99% increase in cancer risk and the authors emphasised that the increased risk was not just limited to lung carcinogenesis.

Peripheral blood leukocytes obtained from 15 uranium miners with radon exposures ranging from 10 to 5400 WLMs have demonstrated a significant increase in a number of CAs including chromatid gaps and breaks, and the formation of dicentric rings in miners compared to controls [163]. Despite this, no consistency was observed between exposure and aberration frequency. An additional follow-up study [164], which also assessed peripheral blood lymphocytes, investigated five groups of radon exposed miners from <100 to >3000 WLMs (*n* = 100, including controls). In general, CAs increased with exposure with the exception of the highest studied concentrations (>3000 WLM) where there was a discernible decrease of deletion prevalence, which was independent of any confounding factors. Nevertheless, they do conclude that CAs represent a sensitive marker of radiation exposure.

Significant increases in CA frequency, MN formation and SCEs have been observed in miners exposed to high radon concentrations when compared to two control groups [165,166]. A cohort of German uranium miners [167] also exhibited increased CAs in their lymphocytes, along with increased MN formation in their lung macrophages and an increased prevalence of fibronectin and the cytokine tumor necrosis factor-alpha (TNF-α) in their bronchoalveolar fluid. Furthermore, two studies of radon exposed miners from the Czech Republic also found an increase in chromatid breaks and MN in miners from a number of different mines [168,169], although in one of the groups no significant difference was observed for increased dicentric ring formation between the miners and controls. A group of Namibian miners exposed to radon demonstrated a reduction in testosterone levels and neutrophil count, along with a three-fold increase in CAs [170].

Further investigations of blood samples from 165 active underground uranium miners exposed up to 600 WLMs between 1981 and 1985, along with 141 former miners from 1998 to 2002, were analysed for CAs and compared to a control group of 175 seemingly healthy subjects [171]. The data revealed a 7–12-fold increase in aberrations in the active mining group, particularly for acentric fragments. Interestingly, the values for the former miners were not significantly different from those of the active miners, suggesting aberrations can persist for many years after exposure, with even the largest decrease in observed aberrations in the form of acentric fragments still remaining 2–3 times higher than in the unexposed population.

There are therefore numerous examples of radon exposed miners exhibiting increased CAs and this is of concern because of the supposed association between elevated CA frequency and the risk of carcinogenesis.

#### 3.1.2. Investigations of Chromosome Aberrations in Non Mining Individuals

Despite a clear association between increased CA frequencies in radon exposed miners, the risks of domestic radon exposures at lower doses have also been explored using CA analyses. Reports of increased frequencies in CAs have not just been reported for occupational exposures, such as among nuclear workers [172], but also, as discussed below, in large numbers of individuals exposed to background radiation in Brazilian, Austrian, Chinese, German, Russian and Slovenian populations, although some studies have not identified significant associations.

A continuation of the Costa-Ribeiro *et al.* [159] study investigated 202 subjects from a Brazilian population environmentally exposed to high background radiation concentrations [173]. An increase of chromosome breaks was identified in blood lymphocytes of subjects who had a local residency of at least eight-years when compared to a control group of 147 individuals from a region of similar socio-economic status but with normal background levels of radiation. A similar study of individuals from an Austrian population exposed to high levels of background radiation from radon [174] found results in agreement with this previous study, including evidence for a dose-response effect above low exposures, although they did observe a decrease in two-event aberrations in a select group of miners exposed to particularly high levels of radon.

A study of individuals living in high background radiation areas (with exposure levels 2.9-times those of the control areas) in Guangdong Province, China found higher levels of dicentric and ring formation aberrations in lymphocytes compared to controls [175]. A matching epidemiological study, however, found mortality from all types of cancer in the high background radiation areas was lower than in the control areas (including leukemia and breast and lung cancers) although the difference was not statistically significant. Dicentric and ring formations were also significantly increased in lymphocytes of 25 individuals, with known radon concentrations 4 to 60-times the German average of 50 Bq·m^−3^, when compared to controls from an assumed low-level radon area in South Germany [176]. No difference in CA frequency was identified between males and females suggesting the damage did not appear to be gender specific. Nevertheless, despite a clear trend with age, a Finnish study [177] found no correlation between domestic radon concentrations (<100 to >800 Bq·m^−3^) in 84 non-smokers and CAs.

A study that followed 6430 healthy people from across Central Europe [178], for whom chromosomal aberration surveys had been performed between 1978 and 2002, provided further evidence for an association between high CA frequencies in peripheral blood lymphocytes and an increased cancer risk. The study identified a significant increase in cancer risk assessed by the frequency of CAs, with a relative risk of cancer of 1.78 in the medium and 1.81 in the high frequency groups when compared to the low frequency group (relative risk = 1).

Eighty-five Slovenian pupils aged 9–12 years old attending an elementary school with high radon concentrations up to 7000 Bq·m^−3^ were identified as having significantly higher levels of CAs and MN compared to a control group of pupils that were the same age but attended a school with concentrations <400 Bq·m^−3^[179]. Mean CA frequencies in blood lymphocytes of residents, including children and teenagers, from high radon concentration areas in Gornaya Shoria, Russia, were significantly higher than those of controls in both home and school based environments [180–182].

In addition, 38 cave tour guides exposed to an estimated annual dose of 40–60 mSv from background radiation from radon were also identified as having higher frequencies of CAs and MN [183] with the principal abnormality representing chromosomal breaks and acentric fragments.

From the environmental studies identified, such an association between increased radon exposure and higher frequencies of CAs appears to be apparent in non-mining as well as mining cohorts.

### 3.2. Laboratory Chromosome Aberration Investigations

Numerous *in vitro* studies have also considered radon and CA formation. Human peripheral lymphocytes have demonstrated an increase in CAs after *in vitro* exposure to 18 cGy of radon and its progeny [150] with 80% of the CAs being chromatid deletions and another 10%–15% chromatic exchanges. Cells were fixed 4, 11, 14 or 17 h after exposure but some dicentric and ring chromosomes were also observed at later harvest times. A linear increase in CAs was observed for cells exposed to increasing doses harvested at the same time. Interestingly, aberrations were approximately one-half lower at each harvest time if cells were pre-exposed to 2 cGy of X-radiation suggesting an adaptive effect.

Data from irradiated haemopoietic cells with environmentally relevant doses of Pu-238 demonstrated a higher frequency of CAs in clonal descendants [151], providing early evidence for transgene rational instability. In a subsequent study investigating chromosome abnormalities of bone marrow cells from four donors [42], CAs were observed in two of the four individuals and the authors highlighted the causal link between chromosome instability and leukemia.

Sister chromatid exchanges have also been observed in murine 10T1/2, 3T3 cells [152] and CHO cells exposed to a low dose of 0.31 mGy of alpha radiation [153]. An increased frequency of SCEs was observed in 30% of the CHO cells although the authors calculate only 1% of the cells were traversed by an alpha particle, suggesting further evidence of a bystander effect. Similar results have been noted in human lung fibroblast cells [31] with radon doses from 0.4 to 12.9 cGy and a dose-dependent increase was observed at low doses. It was noted that the numbers were larger than predicted, based upon predicted numbers of cellular alpha particle traversals, and suggested that alpha particle irradiation was associated with an extranuclear mechanism in inducing SCEs. A follow up study in the same cell type [32] noted an increase of SCEs in cells that were not directly exposed to alpha particles; instead, culture medium from exposed cells was transferred to the unexposed cells. The authors also noted that the extranuclear SCE-inducing factor, or factors, had to survive freeze-thawing and was heat labile, suggesting it may be a protein [184]. A later report identified an increased intracellular production of superoxide anions and hydrogen peroxide after alpha particle exposure even in cells not directly irradiated [48]. The transmission of effects to non-irradiated lung fibroblast cells after exposure to supernatant of irradiated cells was further confirmed by another study [154]. They also identified the transforming growth factor beta 1 (TGF-β1) cytokine as a mediator of the bystander response and, interestingly, a promitogenic effect with augmentation of cell growth. An increase in the production of the TP53 protein and cyclin-dependent kinase inhibitor 1A (CDKN1A) was detected by Western blotting, along with a decrease in cell division control protein 2 (CDC2). Interestingly, modulation of TP53, CDKN1A, CDC2, CCNB1 and RAD51 have also previously been reported after Pu-238 irradiation in non-traversed bystander cells [155].

Following exposure to bismuth-212, which had been chelated to diethlyenetriamine pentaacetic acid (DTPA) to prevent cell entry or attachment ensuring all exposure was external, CHO cells, along with the mutant derivative radiosensitive xrs-5 cell line that is incapable of repairing X-radiation-induced DNA double-strand breaks, displayed an increase in CAs [149].

CAs have also been observed in blood lymphocytes at very low doses (0.03–41.4 mGy) using polonium-214 derived from a gaseous radon dosing apparatus [156]. Aberration frequency rose by more than a factor of 10 between 0.03 and 0.10 mGy before reaching a plateau at exposures of 0.10–5 Gy. At higher doses a linear dose-effect was observed. This again suggests deviation from the linear-dose response relationship at low-dose exposures.

Increases in the frequency of dicentric and acentric fragments, along with centric ring formations and MN have been reported in irradiated blood samples taken from healthy non-smokers using low doses (0–127 mGy) of radon gas delivered *in vitro* [157]. Another *in vitro* study investigating the synergistic effect between radon and smoking examined CAs in blood lymphocytes of both smokers and non-smokers [158]. Blood samples were exposed to a wide range of concentrations from 0 to 35,643 kBq·m^−3^ using a portable irradiation assembly [185] providing doses between 0.9 and 5.2 mGy. Compared to the non-smokers, the smokers’ lymphocytes showed a significant increase in radon-induced dicentric fragments, acentric fragments and chromatid breaks and it was concluded that the study demonstrated lymphocytes of smokers were more unstable to radon exposure.

It is therefore clear that radon and its progeny can produce CAs of a number of different forms in a variety of cell types *in vitro* in addition to bystander effects. It is important to note however that the evidence of an increased CA frequency and detection of DNA damage in high background radiation regions has not necessarily resulted in an increased cancer incidence in some of the studies, suggesting that the correlation between CA frequency and cancer risk may be complicated by the time dependence of the cytogenetic test output.

### 3.3. Biomarker Chromosome Aberration Ratio Studies

With a consistent observation between increased radiation exposure and an increase in CAs, similar suggestions have been made with regards to the possibility of identifying a biomarker of previous exposure to radiation using CAs. Although many CAs are fatal to the cell, some are maintained past cell division [186–188]. The concept of energy deposition from alpha particles occurring over a smaller area than lower LET radiation has resulted in a number of theoretical concepts to identify past exposure. One such suggestion has been that exposure to high-LET radiation results in a low ratio (F) between intra- (same chromosome), inter-chromosomal (separate chromosomes), and inter-arm exchange-type aberrations [189]. However, the authors state that the F value has not been adequately observed epidemiologically and suggest a theoretically more representational method, termed the H value, with a stronger differential ability, which represents the ratio of inter-CAs, including dicentric or translocations, to intra-arm chromosomal aberrations, including acentric inversions or interstitial deletions [190]. Nevertheless, aberration complexity is still reported as a noteworthy outcome of high-LET radiation [191] and some difficulties still remain in observing differences between intra and inter-chromosomal changes [192].

### 3.4. Experimental Investigations of Micronuclei

Another potential consequence of CAs is the formation of micronuclei (MN). MN result from genotoxic events and form following mitosis as cytoplasmic fragments of chromosomes that have not been incorporated into daughter nuclei [193,194]. A number of studies investigating MN formation following radon exposure have been conducted. Exposure to ionizing radiation can lead to an increase in MN formation [195] and there is some evidence that MN frequency can be used as a biomarker to help predict cancer risk [196] with significant increases in MN frequency having been observed following radiotherapy [88].

Comparisons of MN frequency in rats identified linear increases in frequency following *in vivo* exposure to 0, 115, 213 or 323 WLMs of radon gas or *in vitro* exposure using primary rat lung fibroblasts [197] with cell proliferation appearing to be unaffected as a result of MN induction. The authors calculate that *in vivo* exposure in rats of 1 WLM resulted in the equivalent level of damage induced by 0.79 mGy *in vitro*. Elevated levels of MN have also been observed in the alveolar macrophages of rats exposed to 120, 225, 440 or 990 WLMs, with peak frequencies occurring around 13 days after exposure [198].

CHO-K1 cells exposed to 3.2 MeV alpha particles producing 0–5 cellular traversals demonstrated a linear relationship between the number of hits and MN induction [199] although 72% of the cells contained no MN even after five alpha particle traversals, demonstrating a possible variation in cell population sensitivity and, in light of the strong relationship between MN formation and cell death, many cells without MN may survive despite the substantial number of traversals potentially resulting in an increased cancer risk. Similar conclusions have also been drawn from another study using exact alpha particle numbers in human-hamster hybrid cells [200].

Human bronchial epithelial cells have also been exposed to alpha particles from Pu-238 as a radon surrogate [201]. An increase in MN was detected following exposure to six equal fractionated doses (2–4 Gy, 730–1460 WLMs) comparable to exposures received by uranium miners. Deletions on chromosomes 7 and 9 were observed and mapped to regions containing the tumor suppressor genes *CDKN2A* (which encodes p16^Ink4A^) and *TES* that are often inactivated in some lung, prostate and skin tumors [202–205]. Increases in MN frequency have also been identified in human A549 lung cells after exposure to Po-210 (acting as a radon surrogate) across a dose range of 0–2000 mGy [206].

Such evidence leads to the conclusion that radon exposure *in vitro* can consistently result in MN formation, which can be an indication of increased carcinogenic potential.

### 3.5. Genomic Studies

The data available for genomics studies are limited with reference to direct radon exposure and as a result they will not be elaborated on in detail here. However, a range of effects have been observed following low-LET irradiation including alterations in gene expression [207], microRNA modulation following chronic and acute exposures [208], differential gene expression between irradiation and bystander cells [209], transcriptional modification of mitochondrial genes [210] and a suggestion that relative levels in the expression of mRNA in blood lymphocytes may provide a biomarker of exposure [211], highlighting the importance of genomic investigations when studying the biological outcomes of radiation exposure.

## 4. Implications at Low Doses

### 4.1. Investigations of the Linear, No-Threshold Dose-Response Hypothesis

**Figure 3:**
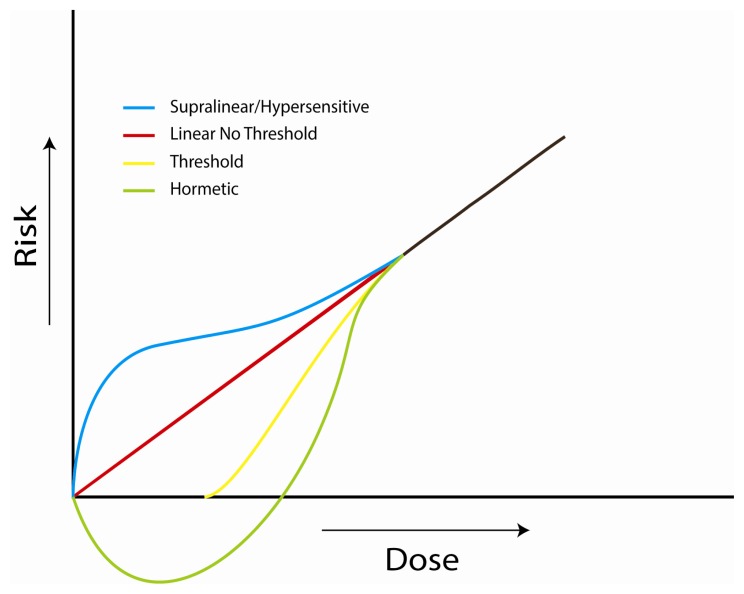
Graphical representation of some of the suggested hypotheses of dose-response functions at low doses, including hormetic, threshold, linear no threshold and supralinear (u-shaped) responses.

The current risk model as recommended by the National Research Council (NRC) in its Biological Effects of Ionizing Radiation Report VII (BEIR VII) [11] is to estimate risk at low exposures on the basis of a linear, no-threshold (LNT) dose-response relationship, whereby potential risk at low doses is calculated by extrapolation from medium and higher doses. The LNT model plays a central role in estimating risk as a result of radiation exposure and is routinely used by various protection agencies [212]. Even so, there is some evidence for a deviation from the LNT model at low doses and a number of alternative hypotheses have been suggested, including threshold, hormetic and supralinear (hypersensitive) biological responses (Figure 3).

There have been suggestions for both a potentially greater or lesser risk than what would currently be predicted based upon the LNT model. It may be possible that at lower doses a number of beneficial cellular mechanisms are induced yielding a protective or hormetic influence [213]. Similarly, hypersensitive responses could result in increased biological damage increasing the risk at low doses [214–216].

Extrapolating risk linearly from medium and high doses implies an effect that is stochastic in its origin, whereby even at the smallest doses an effect would be observed and to decrease the dose would result in a reduction in the number of cells exposed without altering the condition of the insult. The foundations for this mechanism are constructed on the evidence that neoplasms can be of monoclonal origin as well as the observation that a single alpha particle traversal can induce significant cellular damage that can be both permanent and evident in further generations. This is opposed to an effect that would be deterministic in its basis, implying a multicellular foundation whereby a threshold dose may need to be achieved in order to initiate a response and below such a threshold no effect would be observed [217].

### 4.2. Investigations of Bystander Effects

Historically, it has been a widely held belief that any effect resulting from exposure to ionizing radiation is the consequence of nuclear DNA exposure, either by direct irradiation or through radiolysis [218]. However in recent years, some evidence has accumulated suggesting that a number of biological effects relevant to carcinogenesis, which have been credited to nuclear irradiation, can also be produced after cytoplasmic exposure [219,220]. Furthermore, there are now some studies that have reported the induction of biological effects in cells that have not been directly irradiated and where no direct traversal of a charged particle has occurred [221,222]. These effects are referred to as bystander effects and, although their exact mechanisms are yet to be elucidated, there is evidence across a number of studies, with disparate methodologies, for cellular signalling [155,223–226]. Moreover, many bystander effects have been reported at low doses and a response that augments the effects of exposure across large numbers of cells could be a major contributor to the effects observed at low doses. If such bystander effects were also found to occur *in vivo* then the exactitude of the LNT dose-response hypothesis at low doses may have to be called into question.

Some of the identified outcomes that have been attributed to the bystander effect include CAs, including MN formation and SCEs; apoptotic progression and inhibition; modification of gene and protein expression; neoplastic transformation; mutagenesis; cytokine and growth factor production; and generation of γ-H2AX species, indicative of double strand DNA breaks [219,221,222,227–229].

Interest in the bystander effect first occurred after initial investigations by Nagasawa & Little [153] identified SCE induction in CHO cells at alpha particle doses from plutonium-238 as low as 0.31 mGy. While only 1% of cell nuclei were identified as having been traversed, 30% of cells displayed an increased frequency of SCEs. The authors noted that the dose range used is within that expected from domestic radon exposure. Other reports of irradiation of just 10% of a cell population have identified mutant yields similar to when the entire population is irradiated [215].

Since the initial investigations, a number of studies have continued to report bystander effects in cells. Typically, these experiments either use microbeams, emitting single particles, or cell media are transferred from irradiated to nonirradiated cells. There is some debate with regards to the exact mechanisms these two techniques are investigating and a number of extensive reviews explore these [214,230–233].

In contrast to some of the studies that have observed deleterious outcomes as a result of the bystander effect, protective and/or adaptive responses have also been observed in bystander cells including reduced radio-sensitivity [234,235] and increases in the elimination of abnormal cells reducing neoplastic potential [236], which could provide adaptation against future exposures and carcinogenic development in mutated cells [219]. The current mechanistic understanding of the potential for bystander effects to have both deleterious and beneficial effects following irradiation is evidently complex and further research into this area is vital.

### 4.3. Investigations of Adaptive Responses

Not to be confused with hormesis, a potentially beneficial adaptive response has been observed whereby the effects of larger doses of irradiation are minimised when preceded by a previous non-lethal dose [237,238]. The effect has been described in a variety of cell types and it has been suggested to account for the lack of expected cancer incidences in high background radiation areas.

An example of one study suggesting an adaptive response is from Ramsar, Iran, where many people receive very high doses of background radiation from radon of up to 260 mSv·y^−1^ and many individuals have lived in these conditions for generations [239]. A reduced CA frequency following *in vitro* exposure of lymphocytes from individuals living in the Ramsar area to 1.5 Gy of gamma radiation was observed when compared to lymphocytes from people in normal radiation exposure background areas, suggesting a possible adaptive response in these individuals. It is evident that such a response appears to contrast directly with many of the previously discussed miner and laboratory based studies, for which there are a much greater number, where CA frequency increases after exposure and the reasons for this remain unclear.

It is clear that the bystander effect and any potential deviations from the LNT dose-response hypothesis propose implications for risk estimates at low doses, whereby the primary factor affecting cellular response may not be principally defined by dose [240]. However, difficulty in identifying whether cancer risk is increased or decreased at low doses has lead to the decision of the BEIR VII Committee to maintain the LNT model. The report states that it is unclear if the influence of the bystander effect would result in an overall positive or negative health effect and they conclude that the linear, no-threshold dose-response relationship remains generally in line with the evidence available at this time [11]. It may, therefore, still represent the most accurate method to assume the level of risk currently available [217].

## 5. Discussion

There is substantial evidence that exposure to radon and its progeny is the second leading cause of lung cancer behind smoking [45] and concerns have been raised regarding its potential to induce other neoplasms including leukemia [41,241,242] and non-melanoma skin cancer [21,40,243].

Ionizing radiation from radon and its daughter products can induce a variety of cytotoxic and genotoxic effects that are known to be mutagenic and increase carcinogenic potential. Such effects can include genetic mutations, generation of reactive oxygen species, modification of cell cycle regulation (e.g., mitotic delay and inhibition of apoptosis), cytokine up and/or down regulation, CAs and MN formation and generation of γ-H2AX species (indicative of double-strand DNA breaks) [244,245]. These effects can vary depending upon a number of different factors including but not limited to dose, frequency of dose, cell type, cellular conditions (such as cell-cycle stage during exposure) and intra and inter signaling between neighbouring cells [10,246–248].

Identification of a specific genetic response to radon exposure would provide significant assistance to the elucidation of radon-induced carcinogenesis and could act as both a useful biodosimeter and an identifier of risk at typical exposures. Despite promising early investigations [72], it now appears evident that such a mutation hotspot is not located at the codon 249 region of the *TP53* gene. Other regions, such as at the *HPRT* locus, also demonstrate large variability in the current literature. This may best be explained as a result of the lack of knowledge with regards to exposures at low doses whereby expected outcomes appear to deviate from the LNT hypothesis recommended by the BEIR VII Committee. Many of the biomarker studies also appear to suffer from relatively small sample sizes, which may potentially explain why some of the results are inconsistent. Despite the current lack of experimental agreement for identifying a hotspot biomarker, the possibility of identifying such a unique marker should not be ruled out. More investigations into a consistent genetic modification as a result of radon exposure are therefore required. Nevertheless, it is clear that if potential molecular biomarkers can be identified despite variation at low doses, they should be used with caution when attempting to estimate exposure or risk.

CAs (including insertions, deletions, translocations, SCEs, ring formation, duplications, inversions and formation of MN) are thought to significantly increase the risk of neoplastic progression and to intensify carcinogenic capacity. Such cytogenetic markers of biological damage have been consistently observed both *in vitro* and *in vivo* in a large number of studies following exposure either to radon and its progeny or a surrogate alpha particle emitter used in its place for comparison.

Regardless of stochastic or deterministic foundations, it appears that the current evidence is too varied to conclude with certainty whether or not the LNT model holds true at low doses and this is particularly pertinent in light of implications proposed by intercellular communication such as those attributable to bystander effects. However, the possibility of underestimating the carcinogenic risk of radon exposure is likely to be increased when considering the additional influence of a “bystander” effect generated by inter and/or intra-cellular signaling mechanisms, the details of which remain largely unidentified. It is evident however that cytogenetic effects can be observed in non-irradiated cells and further research efforts are required to elucidate the details of these mechanisms of damage [249].

## 6. Conclusions

Exposure to radon and its progeny can induce a range of cellular and molecular cytogenetic effects both *in vitro* and *in vivo*. Data from animal, laboratory and miner studies, as well as residents of the domestic home and school environments, have all demonstrated the potential of radon exposure to induce biological damage. When taken together, these data suggest that exposure to radon and its progeny (when concentrations are elevated at medium and high doses, such as those that exceed the recommended action levels) can represent a significant public health risk. Future work, particularly in the low and ultra-low dose range where data for cellular responses are varied, is also vital to elucidate the full risk from the ionizing radiation experienced by the general population through natural environmental radon exposure.

## Figures and Tables

**Table 1:** Energy emissions (MeV) of the radon-222 decay series.

Isotope	Emission energy (MeV)	Decay type
Radon-222	5.49	α

Polonium-218	6.00	α

Lead-214	0.67, 0.73	β
	0.35, 0.30, 0.24	γ

Bismuth-214	1.54, 1.51, 3.27	β
	0.61, 1.76, 1.12	γ

Polonium-214	7.69	α

Lead-210	0.06, 0.02	β
	0.05	γ

Bismuth-210	1.16	β
	0.27, 0.30	γ

Polonium-210	5.30	α

Lead-206		Stable

**Table 2:** Estimated lifetime risk of lung cancer death by radon level for never smokers, current smokers and the general population assuming lifetime exposure (adapted from United States Environmental Protection Agency [55]).

Radon level Bq/m^3^	Lifetime risk of lung cancer death from radon exposure in homes (%)		

	Never smokers	Current smokers	General population
740	3.6	26.3	10.5
370	1.8	15	5.6
296	1.5	12	4.5
148	0.7	6.2	2.3
74	0.4	3.2	1.2
46.25	0.2	2	0.7
14.8	0.1	0.6	0.2

**Table 3:** Key human *TP53* analyses of exposure to radon or its surrogates.

Exposure a	Cancer/cell type	G to T hotspot transversion	Number of observed mutations/number of cancers studied	Exon(s) sequenced	Reference
-	NS	Not evident	7/19	5–9	[60]
1382 WLM	11 LCC; 41 SCC	(16/29)	29/52	5–9	[62]
-	23 AC	Not evident	0/23	5–9	[63]
4 Gy (~1460 WLM)	NHBE cells	Not evident	-	7	[69]
~1160 WLM (estimated mean)	19 AC; 19 SmCC; 9 SCC; 2 Mixed; 1 ASC	Not evident	5/50	7	[65]
<50 or >140 Bq·m^−3^	73 SmCC; 59 SCC; 86 AC; 25 Other	Not evident	58/243	5–8	[70]
~1100 WLM	19 AC; 19 SmCC; 9 SCC; 3 Mixed	Not evident	5/50	7	[66]
-	16 SCC; 11 AC; 1 SmCC; 1 LCC	Rare (1/29)	12/29	5–7	[68]
-	29 SCC	Rare (2/29)	Not reported/29	7	[67]
11.1–51.8 Bq·m^−3^	NS	Not evident	0/4 (Only 4/16 could be analysed)	4–8	[71]

a Exposures are based on presented values;

Abbreviations: NS (not specified); LCC (large cell carcinoma); SCC (squamous cell carcinoma); AC (adenocarcinoma); NHBE (normal human bronchial epithelial); ASC (adenosquamous carcinoma).

**Table 4:** Key cytogenetic analyses of exposure to radon or its surrogates *in vitro* with identified doses.

Exposure	Cell type	Dose range	Dose rate	Energy (MeV)	Investigated abnormality	Reference
212Bi	CHO-K1; xrs-5	1–5 Gy	0.125–0.5 Gy/h		CellSurvival; CAs; HPRT mutations	[149]
222Rn	Blood lymphocyte	2–18 cGy	15 cGy/h		Chromatid deletions	[150]
238Pu	Multipotential murine marrow	0.25–1 Gy	-	3.3	CAs	[151]
238Pu	Multipotential human marrow	0.25–1 Gy	-		CAs	[42]
238Pu	10T1/2; 3T3	2.5–5 cGy	-	5.3	CAs; SCEs	[152]
238Pu	CHO	0.16–4.9 mGy	0.147 Gy/min	3.7	SCEs	[153]
238Pu	HFL1	0.4–12.9 cGy	3.65 cGy/s	3.5	SCEs	[31]
238Pu	HFL1	1.8–12.9 cGy	3.65 cGy/s	3.5	SCE	[32]
238Pu	HFL1	0.4–19 cGy	3.65 cGy/s	3.5	ROS generation	[48]
238Pu	HFL1	1–19 cGy	3.65 cGy/s	3.5	ROS, TP53, CDC2, CDKN1A and TGF-β1 generation; cell proliferation	[154]
238Pu	AG1521; AG1522; GM5758; GM6419; GM8333	0.3–75 cGy	9.9 cGy/min	3.65	TP53, CDC2, CDKN1A, RAD51 and CCNB1 generation	[155]
214Po	Blood lymphocyte	0.03–41.4 mGy	-	7.68	CA	[156]
4He	HBPE	0.6 Gy	-	4.0	Colony formation; malignant transformation; TP53 mutations; CA; invasion ability	[76]
222Rn	Blood lymphocyte	0–127 mGy	-	5.5	CA; CBMN	[157]
222Rn	Blood lymphocyte	0.9–5.2 mGy	-	5.5	CA	[158]

Abbreviations: CA (Chromosome/chromatid aberrations); SCE (Sister chromatid exchanges); ROS (Reactive oxygen species); CBMN (Cytokinesis blocked micronuclei).
